# Protein Kinase A in neurological disorders

**DOI:** 10.1186/s11689-024-09525-0

**Published:** 2024-03-13

**Authors:** Alexander G. P. Glebov-McCloud, Walter S. Saide, Marie E. Gaine, Stefan Strack

**Affiliations:** 1 Department of Neuroscience and Pharmacology, Bowen Science Building, University of Iowa, Carver College of Medicine, 51 Newton Road, Iowa City, IA 52242 USA; 2 Department of Pharmaceutical Sciences and Experimental Therapeutics, College of Pharmacy Building, College of Pharmacy, University of Iowa, 180 S. Grand Ave, Iowa City, IA 52242 USA; 3 Iowa Neuroscience Institute, Intellectual and Developmental Disabilities Research Center, Iowa City, IA USA

**Keywords:** PKA, cAMP, Protein phosphorylation, CREB, Gene transcription, MAPK, Kinases, Neurodevelopment, Learning, Memory, Cognition, Endocrine Systems, Metabolic Disorders, Movement Disorders, Neurodegeneration

## Abstract

Cyclic adenosine 3’, 5’ monophosphate (cAMP)-dependent Protein Kinase A (PKA) is a multi-functional serine/threonine kinase that regulates a wide variety of physiological processes including gene transcription, metabolism, and synaptic plasticity. Genomic sequencing studies have identified both germline and somatic variants of the catalytic and regulatory subunits of PKA in patients with metabolic and neurodevelopmental disorders. In this review we discuss the classical cAMP/PKA signaling pathway and the disease phenotypes that result from PKA variants. This review highlights distinct isoform-specific cognitive deficits that occur in both PKA catalytic and regulatory subunits, and how tissue-specific distribution of these isoforms may contribute to neurodevelopmental disorders in comparison to more generalized endocrine dysfunction.

## Background

Regulation of signal transduction by phosphorylation is one of the most well studied post-translational modifications to date. Seminal work from many laboratories identified phosphorylation to be a critical form of regulation in neuronal signaling in response to Ca^2+^ transients [1–4]. The dependence of this phosphorylation on cyclic adenosine 3’,5’ monophosphate (cAMP) would help differentiate Protein Kinase A (PKA) from Ca^2+^/Calmodulin-dependent protein kinase II (CaMKII) as critical Ca^2+^-sensitive messengers in the brain [5, 6]. It is now well established that PKA plays a central role in the induction of both functional and structural changes in neurons such as long-term potentiation (LTP), and long-term depression (LTD)  [7–9] (For reviews see Abel and Nguyen 2008 and Christensen and Nairn 2021 [10, 11].

In addition to signal transduction in the adult brain, there is emerging evidence that suggests PKA plays a critical role in neurodevelopment, and pathogenic variants of PKA can produce neurodevelopmental disorders that impair learning and memory, cognition, motor function and coordination, pain sensing, and social behavior. In this review, we will discuss the known genetic risk factors and functional outputs of the cAMP signaling pathway and their contributions to neurodevelopmental disorders.

## PKA structure/function

PKA is a basophilic serine/threonine kinase with broad substrate specificity, regulating diverse signaling pathways from glucose homeostasis to gene transcription. In the native state, the PKA holoenzyme exists as a hetero-tetrameric complex of two catalytic subunits (C subunits) autoinhibited by two regulatory subunits (R subunits). Binding of cAMP to the R subunits promotes a large conformational change that unleashes the C subunits from their dimeric holoenzyme and promotes phosphorylation of downstream targets. Due to its broad substrate specificity, cells take advantage of targeted PKA phosphorylation through subcellular localization and anchoring of the R subunits to discrete compartments by association with A-Kinase Associated Proteins (AKAP’s). Localized phosphodiesterase (PDE) activity further controls cAMP signaling and activation of PKA in discrete compartments within the cell [12–14].

### The catalytic subunit

**Fig. 1:**
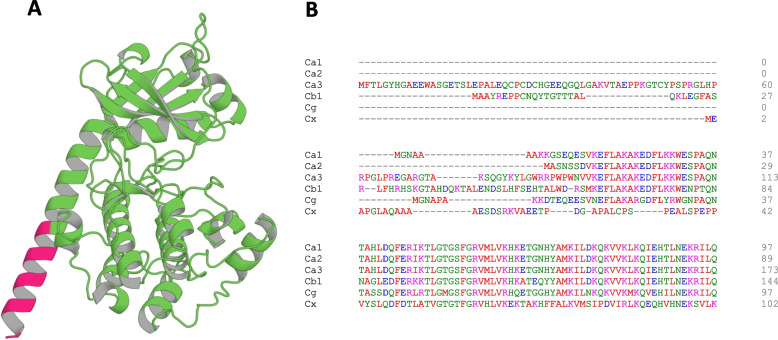
The Catalytic Subunit: **A** Representative AlphaFold Structure of the Cα1 catalytic subunit of PKA. The protein N-terminus (MGNAAAAKGSEQES) is highlighted in hot pink, and the more conserved regions of the catalytic subunit are colored in green. **B** Multiple sequence alignment of the N-terminus for the PKA catalytic subunit isoforms Cα1, Cα2, Cα3, Cβ1, Cγ, and Cχ

Eukaryotic protein kinases have a small N-terminal lobe with 5 β strands and 3 α helices, and a larger, mostly helical, C-terminal lobe, connected through a disordered hinge region that binds Mg^2+^/adenosine 5’-triphosphate (ATP) between them (Fig. 1A). The majority of PKA C subunits expressed in the human body are encoded by two genes – *PRKACA* and *PRKACB*, which gives rise to the Cα and Cβ catalytic isozymes [12–15]. Two other putative PKA C subunit related genes, *PRKACG* and *PRKX* which encode the Cγ and Cχ isozymes, have also been identified, but their biological relevance is currently unclear [16–18]. The gene that encodes the predominant isoform, Cα, is located on the reverse strand of chromosome 19 at p13.1 and is ubiquitously expressed in all tissues [19]. Alternative splicing produces three splice-site isoforms of the Cα subunit, creating Cα1, Cα2, and Cα3. Cα1 is the predominant PKA C subunit and is widely expressed in most tissues. This isoform has a longer N-terminal transcript compared to the Cα2 isoform, which is specifically expressed in the testes [20]. Cα3 is the longest Cα isozyme but has not been widely studied [13, 21, 22] (Fig. 1B).

In contrast, *PRKACB* has four alternative 5’ exons that can produce up to 16 different, catalytically active splice isoforms [12, 23–26]. *PRKACB* shares about 93% sequence homology with *PRKACA* and encode for a structurally and functionally similar catalytic domain core [27]. Like Cα1, Cβ1 is ubiquitously expressed in most tissues [25]. Expression of other *PRKACB* variants are more distinct. Cβ2 is enriched in brain and lymphoid tissues [25, 28], while Cβ3 and Cβ4 are brain specific isozymes [23, 25]. In situ hybridization studies have indicated high levels of expression for Cβ1 in the thalamic areas, dentate gyrus, and pyramidal cells of the hippocampus, while Cβ2 hybridization was more specific to the prelimbic cortex, bed nucleus of the stria terminalis, amygdala, and ventral medial hypothalamic nucleus [23].

### The regulatory subunit

**Fig. 2:**
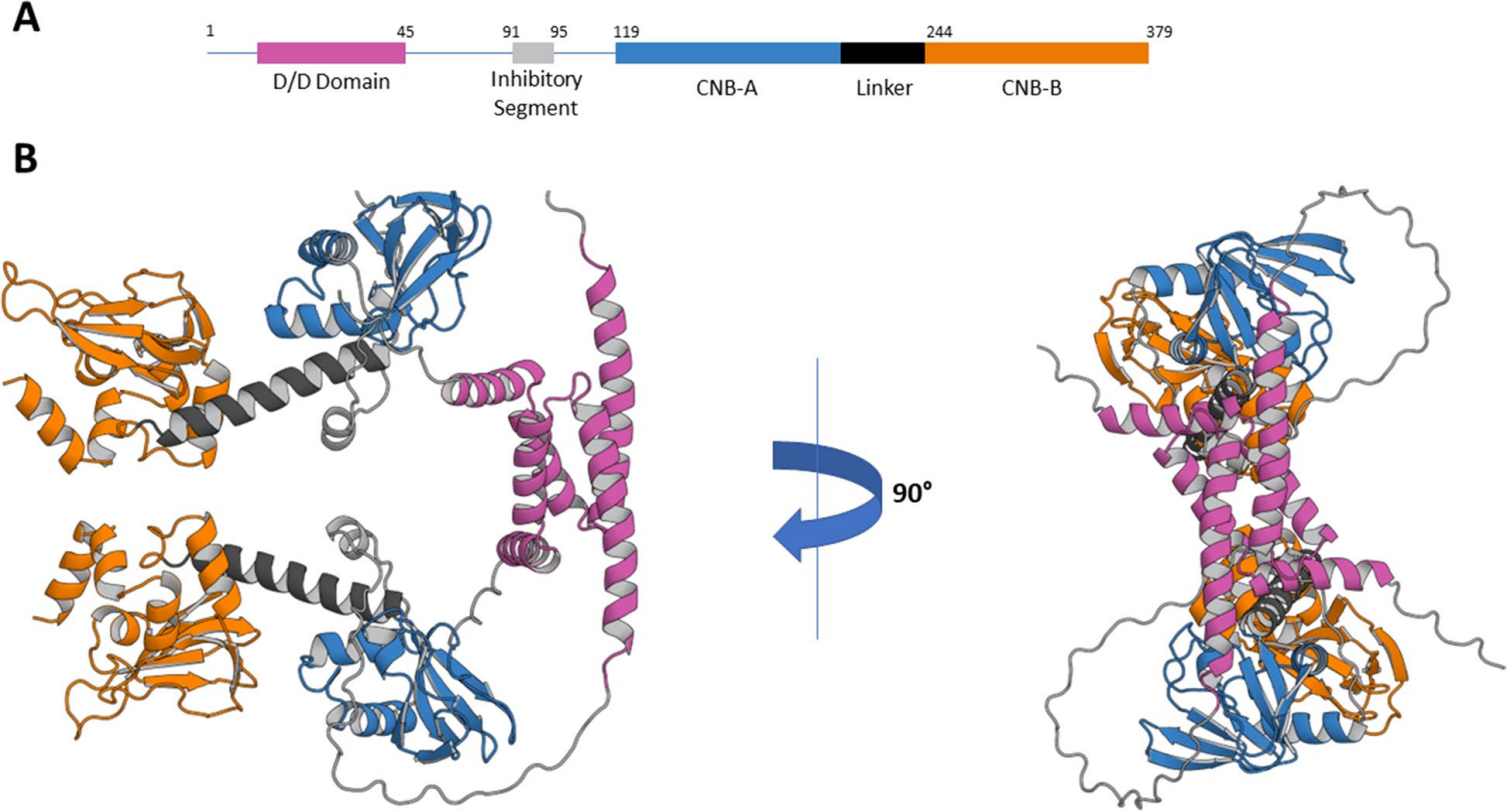
The Regulatory Subunit: **A** Schematic representation of the structural domains encoded by the PKA regulatory subunits. **B** Two representations of the predicted AlphaFold structure of a PRKAR1B dimer. The DD domain is colored in magenta, the inhibitory segment is colored in grey, CNB-A in blue, and CNB-B in orange. The linker region between the two CNB’s is colored in black

The R subunits are encoded by four genes: *PRKAR1A*, *PRKAR1B*, *PRKAR2A*, and *PRKAR2B*. When PKA holoenzymes were first being purified, two major peaks were observed upon elution from strong anion exchange columns and led to the naming of Type I and Type II PKA holoenzymes [29–32]. The α and β isoforms of each Type of R subunit were discovered later through molecular cloning experiments [33–36], resulting in the expression of the RIα, RIβ, RIIα, and RIIβ isoforms. These isoforms all encode for the same general structural features including a Dimerization/Docking (D/D) domain, a small inhibitory segment, and two cyclic nucleotide binding (CNB) domains, typically referred to as CNB-A and CNB-B (Fig. 2A, B). The ratio of Type I and Type II PKA holoenzymes varies among tissues, but many studies suggest that actively proliferating or differentiating tissues express higher ratios of Type I holoenzymes, while terminally differentiated or mature tissues have higher concentrations of Type II holoenzymes (for a review, see Cho-Chung Y. 1995 [37]).

While they all have the same structural features, tissue distribution of the R subunits varies. Both the RIα and RIIα subunits are ubiquitously expressed in most tissues [33, 35]. In contrast, the RIβ subunit is primarily expressed in the brain [38] and the RIIβ subunit is expressed in the brain, endocrine tissues, fat, and reproductive organs [39–41].

### The docking/dimerization domain

As the name suggests, the D/D domain serves two important roles for the PKA holoenzyme: dimerization of two regulatory subunits and “docking” of the PKA holoenzyme into specific subcellular compartments by binding to an amphipathic α-helix in AKAPs [42, 43]. AKAP specificity to R subunits is mediated by amino acids that occupy the groove formed within the D/D domain [42], which allows for PKA C subunits to be compartmentalized in a cell/tissue dependent manner for highly specific cAMP signaling to occur.

While the α and β isoforms of Type I and Type II R subunits are known to form isoform-specific heterodimers, there appears to be no significant evidence for heterodimerization between Type I and Type II R subunits. Additionally, holoenzymes prefer to form homodimers of α and β R subunits of the same isoform [44–46]. The bias toward homodimerization of α and β subunits within each type may provide an additional layer of PKA signaling regulation in a tissue-dependent manner based on relative expression levels of these isoforms.

### The cyclic nucleotide binding domains

For PKA, each R subunit contains two CNB’s, often denoted as the A-site for the more N-terminal lobe and the B-site for the more C-terminal lobe. These two sites have drastically different affinities for cAMP, with the A-site having faster on and off rates of cAMP compared to the B-site. The two CNB’s form an allosteric network that provides a basis for cooperative activation of PKA by cAMP. Upon binding cAMP to the A-site, a large conformational change between the two CNB’s occurs that allosterically modulates binding of cAMP to the B-site. Phosphorylation of downstream PKA targets can only occur after cAMP binds to both the A- and B-sites to induce dissociation of the C subunits from the R subunits. The general conformation of the A and B-sites are drastically different in crystal structures of Type I and Type II R subunits, which may provide a structural basis for differences in Type I and Type II holoenzyme cooperativity and sensitivity to activation by cAMP [47–49].

## Activation of PKA by GPCR’s

**Fig. 3:**
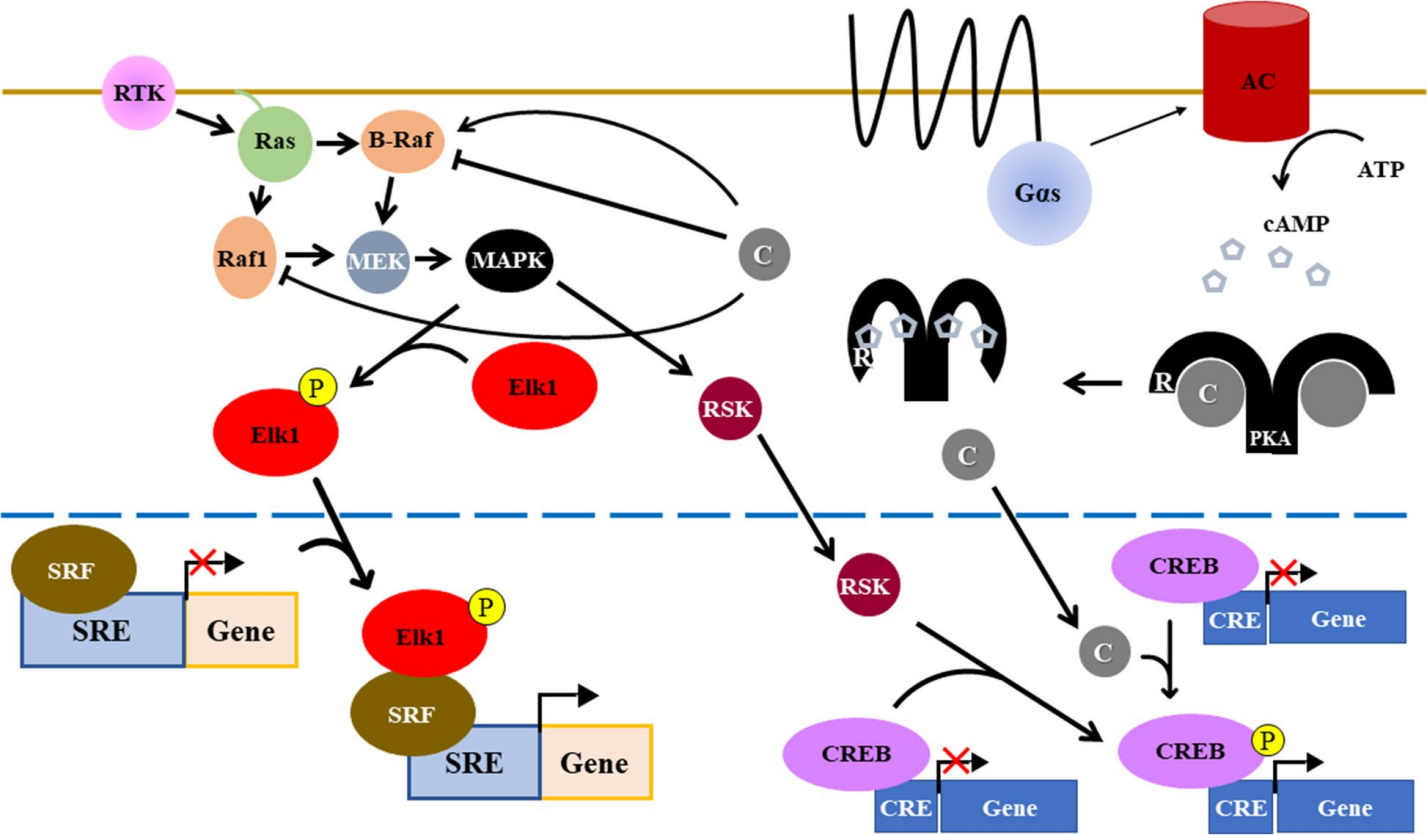
Common PKA signaling pathways: Diagram of the MAPK and PKA pathways. Signaling events leading to Elk1-mediated transcription are shown on the left, while those leading to CREB-mediated transcription are shown on the right. Depending on cell context, PKA either positively or negatively regulates Ras-MAPK signaling. Moreover, MAPK indirectly stimulates CREB-mediated transcription via activation of ribosomal protein S6 kinases (RSKs)

Neuronal function primarily responds to two main mechanisms – fast synaptic transmission through ionotropic receptors and slow synaptic transmission through metabotropic or G protein-coupled receptors (GPCR’s). GPCR’s make up one of the largest families of transmembrane proteins with > 800 GPCR’s identified to date, more than 90% of which are expressed in the brain [50]. These receptors help cells respond to changes in their extracellular environment and induce biochemical signaling pathways upon activation by their specific ligands, as is the case in synaptic transmission. GPCR’s are composed of a heterotrimeric G protein complex coupled to a transmembrane receptor where the G protein complex contains Gα, Gβ, and Gγ subunits [51–53]. The Gβ and Gγ subunits constitutively dimerize and form a trimeric complex with Gα subunits that are bound to guanosine 5’-diphosphate (GDP). Binding of the appropriate ligand to the GPCR’s transmembrane receptor promotes a conformational change in the G proteins that catalyzes the exchange of GDP for guanosine 5’-triphosphate (GTP) within the Gα subunit, and in turn leads to the dissociation or rearrangement of the Gα subunit and the Gβγ dimer [54–56]. Both the Gα subunit and the Gβγ dimer can then bind to downstream targets and initiate their unique biochemical pathways. Signaling through Gα_s_ initiates the conversion of ATP to cAMP by Adenylyl Cyclases (AC’s), which in turn activates proteins with CNB’s such as PKA (Fig. 3). cAMP binding to the R subunit unleashes the C subunit for downstream phosphorylation to occur. Once AC’s become activated by GPCR’s, intrinsic GTPase activity of the Gα subunit hydrolyzes GTP to GDP and dissociates the Gα subunit from the AC, thus ending the signal induced by the initial ligand binding event.

## PKA’s role in learning and memory

Some of the first studies implicating a role for PKA in learning and memory were performed in Aplysia. Aplysia have a gill and siphon withdrawal reflex that can be sensitized following repeated stimulation [57]. Given Aplysia’s relatively simple nervous system, this allows for a comprehensive dissection of the mechanisms underlying this response [58]. Specifically, this sensitization requires long-term facilitation (LTF), the growth of new synaptic connections for neurons involved in Aplysia’s withdrawal reflex. Induction of LTF in cultured Aplysia sensory and motor neurons could be stimulated with the application of serotonin (5-HT) [59, 60], which increases cAMP levels in cells to activate PKA [61].

Early studies using this system discovered that LTF induction requires gene transcription and translation, as inhibitors of both RNA and protein synthesis prevented 5-HT-induced increases in excitatory post-synaptic potentials (EPSPs) [59]. Once activated by cAMP, the PKA C subunit can translocate to the nucleus, where it phosphorylates cAMP response element-binding protein (CREB) [62]. In neurons, CREB is constitutively bound to the cAMP responsive element (CRE) promoter [63, 64]. Following phosphorylation, CREB initiates transcription of genes associated with the promoters it is bound to [58]. In Aplysia sensory neurons, Dash and colleagues found that injection of CRE oligonucleotide sequences prevented induction of LTF following 5-HT stimulation [65]. A greater understanding of the mechanism underlying LTF in Aplysia was uncovered by introducing mutations into CREB. Specifically, CREB Ser119, the residue required for PKA- or CaMKII-dependent initiation of transcription, was mutated to an alanine to prevent CREB phosphorylation. 5-HT treatment of Aplysia neurons expressing the CREB Ser119Ala mutant showed no induction of LTF [66]. To determine which protein phosphorylated CREB, Kaang and colleagues generated a CREB mutant that lacked the PKA phosphorylation motif but still had the CaMKII phosphorylation motif. Expression of this mutant CREB also prevented LTF induction, suggesting that PKA, not CaMKII, phosphorylated CREB at Ser119 [66]. Finally, Bacskai and colleagues showed that the PKA C subunit moved into the nucleus of neurons following elevation of cAMP levels resulting from forskolin, IBMX, and 5-HT stimulation [67]. These results support the idea that the cAMP pathway plays a role in LTF.

While they showed that PKA and CREB were important in memory, the experiments in Aplysia did not provide information about where PKA and CREB act to regulate LTF/long-term memory (LTM). Location-specific information about where LTM is regulated was only acquired with electrophysiology and behavioral data collected from transgenic mice expressing an inhibitory form of PKA RIα (R(AB)) [68]. Electrophysiological recordings of hippocampal area CA1 slices from R(AB) mice revealed deficits in the late phase of long-term potentiation (L-LTP) relative to slices from wild-type (WT) mice [68]. These mice also performed worse than their WT counterparts in the Morris water maze, indicating that they had deficits in spatial memory [68]. Altogether, the experiments in Aplysia and mice reveal that PKA plays a significant role in learning and memory and that these processes appear to be evolutionarily conserved across species.

##  MAPK signaling

 Previous work has shown that PKA regulates the mitogen-activated protein kinase (MAPK) /extracellular signal-regulated kinase (ERK) signaling pathway. Specifically, Li and colleagues showed that PKA phosphorylation of B-Raf Ser365 inhibits ERK signaling by blocking binding of B-Raf to Ras [69]. Other mechanisms of negative regulation of Ras-MAPK signaling have also been reported [70, 71]. However, while this phosphorylation event disrupts B-Raf binding to Ras, PKA can stimulate ERK signaling by phosphorylating different proteins in the ERK pathway in a cell context dependent manner [72–76]. ERK signaling can be stimulated through Rap1 binding to B-Raf, but this interaction is blocked by PKA phosphorylation [77]. However, if PKA also phosphorylates Rap1, B-Raf can indirectly bind to Rap1 by binding to kinase suppressor of Ras (KSR), which itself binds to phosphorylated Rap1 [77].

 The aforementioned PKA phosphorylation events in the ERK signaling pathway are important because of Elk1 activation by MAPK/ERK. Elk1 is a transcription factor expressed in neurons that plays a role in differentiation, cancer, inflammation, and cell growth [78]. Once phosphorylated by ERK, Elk1 translocates from the cytoplasm into the nucleus, where it binds serum response factor (SRF) at the serum response element (SRE) on DNA [79]. Elk1 can also recruit CREB binding protein (CBP) to DNA, which can acetylate histones on specific regions of DNA to promote gene transcription [78]. Elk1 activation also promotes NMDA-dependent LTD in vivo [80] and is associated with taste memory [81] and one-trial avoidance learning [82].

## PKA variants in neurodevelopmental disorders

Pathogenic variants that contribute to some forms of a neurodevelopmental disorder (NDD) have been identified in both PKA catalytic and regulatory subunits. Some of these variants cause disorders that are not primarily associated with neurodevelopment, such as acrodysostosis, but can result in intellectual disability (ID) as a secondary phenotype in a small portion of affected patients. Other pathogenic variants have been shown to directly cause ID with few, if any, secondary phenotypes. An overview of these protein variants and the disorders they cause is described below.

### PRKACA and PRKACB

Pathogenic somatic variants of *PRKACA* often manifest as endocrine and metabolic disorders such as Cushing’s syndrome, cortisol-producing adenomas (CPA’s), hypothalamic hamartomas, and cardiac myxomas [83–88]. Between 23 and 67% of CPA’s can be attributed to a L205R somatic variant in PKA Cα, resulting in constitutive activation of PKA Cα and altering subcellular localization by decreasing interaction with AKAPs [87–91]. Several residues proximal to L205 on PKA Cα localize to a catalytic domain “hot spot” commonly mutated in patients with CPA’s [86–88, 90, 91]. This hot spot alters the structural determinants of the catalytic domain’s specificity for both autoinhibition by R subunits and substrate specificity. Phosphoproteomic analysis of common *PRKACA* and *PRKACB* variants associated with CPA’s identified a profound shift in PKA’s phosphorylation profile, with consistent hyperphosphorylation of citron rho-interacting kinase (CIT), the mitochondrial import receptor subunit TOM34, histone H1.2, and histone H1.4 by three activating *PRKACA* somatic variants [92].

Both pathogenic somatic and germline variants in either *PRKACA* or *PRKACB* were discovered in patients with several congenital abnormalities such as atrioventricular septal defect (AVSD) and polydactyly [93]. Of the seven patients described, two patients with de novo PKA Cβ H88R or H88N mutations had mild-to-severe ID, anxiety, autistic features, and medically refractory focal epilepsy [93]. Using bioluminescence resonance energy transfer (BRET), Palencia-Campos et al. demonstrated the H88 mutants rapidly dissociate from the R subunits upon stimulation with Forskolin/IBMX and have slower reassociation kinetics to all R subunit isoforms compared to WT. Molecular Dynamic simulations suggest the H88 mutations likely disrupt the glycine-rich loop of the catalytic domain N-terminus, which packs onto the gamma-phosphate of ATP during phosphotransferase activity [94]. These models were corroborated by Fluorescence Polarization experiments that demonstrated a significant decrease in binding affinity for a FAM-labeled PKA inhibitor IP20 when varying H88R PKA Cβ at 1 mM ATP, concomitant with a decrease in ATP binding affinity at 5 nM H88R PKA Cβ, but independently of any changes in cAMP affinity [93]. These results suggest that perturbations in the catalytic domain that coordinate ATP binding destabilize substrate binding affinity and autoinhibition.

### PRKAR1A

Pathogenic germline nonsense insertion/deletion variants of *PRKAR1A* are the most common genetic causes of Carney complex, a hereditary condition associated with spotted skin pigmentation, cardiac myxomas, and endocrine tumors [95, 96]. Nonsense-mediated decay of mRNA decreases total expression of the RIα subunit, resulting in constitutive activation of PKA C subunits throughout the body [97].

Similarly, researchers identified several individuals with de novo pathogenic germline variants in *PRKAR1A* that had acrodysostosis, an autosomal dominant disorder characterized by hormone resistance, brachydactyly, craniofacial abnormalities, and short stature [98–101]. Unlike Carney complex, many of the pathogenic variants in *PRKAR1A* that are found in patients with acrodysostosis may lead to decreased sensitivity to cAMP, resulting in a dominant negative effect on PKA activity (reviewed in Michot 2018 [98]). Several patients with acrodysostosis have been reported with mild-to-severe intellectual disability or mental retardation as a comorbidity [102, 103], but the cooccurrence of ID with *PRKAR1A* variants is relatively low. In a recent report, only 1/9 acrodysostosis patients with *PRKAR1A* variants were comorbid with ID [98]. Thus, clinical manifestation of ID is more closely associated with decreased PKA activity through decreased sensitivity of RIα to cAMP. It should be mentioned that while many *PRKAR1A* variants have been identified in Carney Complex, so too have variants in the phosphodiesterases PDE11A and PDE8 which more commonly result in ID, suggesting cAMP concentrations in distinct compartments or cell types may contribute to ID more strongly than total regulation of PKA activity by the RIα subunit [98].

### PRKAR1B

Although RIβ is relatively understudied in comparison to the other R subunits, it has been associated with numerous neurological phenotypes. In 2014, a novel rare pathogenic germline variant in *PRKAR1B* (L50R) was identified by Wong et al. in a three generational family [104]. Twelve members of the family had a neurodegenerative disorder with dementia, frontotemporal dementia (FTD)-like and Parkinsonism symptoms, and the L50R germline variant segregated within the family in affected members. Additional evidence from this study supporting an association between *PRKAR1B* and neurodegeneration was the accumulation of *PRKAR1B* in neuronal inclusions in the affected cases. The age of onset was between 45 and 64 years old and the most common symptoms noted were behavioral changes including self-neglect and delusions, anxiety, memory problems, and motor deficits. Wong and colleagues predicted that the L50R variant altered *PRKAR1B* binding, and therefore the PKA interactome, due to its location in the D/D domain. Notably, no pathogenic *PRKAR1B* variants were found in a cohort of Parkinson’s Disease, neuronal intermediate filament inclusion disease (NIFID) or FTD [104, 105], suggesting this may be a novel and rare variant. A subsequent genetic study of the *PRKAR1B* gene identified 7 variants in FTD and Alzheimer’s disease (AD) patients but these were not predicted to be pathogenic [106]. In contrast to some of the other studies mentioned, common variants located just downstream of *PRKAR1B* have been associated with late-onset AD and present in a punctate formation [107]. Importantly, the Agora database has ranked *PRKAR1B* with a score of 4.43, which suggests a high relevance between *PRKAR1B* and AD based on published evidence including a significant reduction in RNA expression in the temporal cortex and a significant reduction in protein expression in the dorsolateral prefrontal cortex ( https://agora.adknowledgeportal.org/genes ). Overall, these findings suggest an important role for *PRKAR1B* in neurodegeneration and AD.

Recent exome sequencing studies have identified 13 individuals in 2 cohorts with novel pathogenic variants of *PRKAR1B* that leads to global developmental delay, ID, and autism spectrum disorder (ASD) known as Marbach-Schaaf Neurodevelopmental Syndrome (MASNS) [108, 109]*.* Interestingly, 11/13 individuals identified with MASNS had a c.1003C > T missense variant of *PRKAR1B*, which resulted in an R335W RIβ subunit of PKA. The two other patients in the cohort were identified with c.586G > A (E196K) and c.500_501 inv (Q167L) variants. These variants can be found in the R subunit CNB’s, and R335 can be seen making electrostatic contacts with the phosphate group of cAMP in crystal structures, and thus may perturb cAMP binding to the R1β subunit [110].

Many neurologic anomalies are associated with MASNS, including delayed development of fine motor skills, dyspraxia, clumsiness, tremor, dystonia, and congenital hypotonia [108, 109]. Behavioral abnormalities in MASNS patients usually observed in the context of ASD include self-harm, hand/arm flapping, and repetitive, sensory-seeking behavior. Many of these patients also experienced hyperactivity or restlessness, some of whom were formally diagnosed with attention-deficit/hyperactivity disorder (ADHD).

The authors identified *PRKAR1B* to be highly expressed in the pituitary, diencephalon, mesencephalon, and hypothalamus in human embryos and Carnegie stage 22, suggesting a critical role for the RIβ subunit during the development of the embryonic brain. However, no significant abnormalities in brain structures were detected in children with MASNS using magnetic resonance imaging (MRI) or electroencephalography (EEG).

Interestingly, a phenotype unique to the R335W variant, but not the Q167L or E196K variants, was an inherent insensitivity to pain. Parents of children with R335W MASNS have informally reported an intrinsic lack of pain sensitivity during self-injuring behaviors or when holding hot beverages but had normal temperature perception.

Transient expression of R335W RIβ in HEK293 cells has shown both low basal and stimulated cAMP stimulated PKA activity compared to WT (Glebov-McCloud et al., unpublished), suggesting a lack of cAMP sensitivity for PKA activation in mutant RIβ. Thus, PKA signaling in both developing neurons and primary sensory neurons may be impaired by R335W RIβ PKA holoenzymes.

## Conclusion

Taken together, these data provide valuable insights into the role of cAMP/PKA signaling during neurodevelopment. Many of the variants in the cAMP/PKA signaling pathway that are more widely expressed throughout the body to contribute to broader morphological, metabolic, and endocrine dysfunction. This is highlighted by the prevalence of Cushing’s Syndrome and acrodysostosis for patients with *PRKACA* or *PRKAR1A* variants, respectively. However, variants of genes that are enriched or specifically expressed in brain tissues, such as *PRKACB* or *PRKAR1B*, appear to produce more cognitive deficits, as is the case with MASNS. Furthermore, inactivating variants of either C or R subunits, producing a dominant negative phenotype on catalytic PKA activity, appear to manifest as cognitive or behavioral deficits more so than activating variants. A PDE4 inhibitor Zatomilast® is currently in Phase III Clinical Trials for the treatment of cognitive deficits produced by Alzheimer’s disease and Fragile-X Syndrome, further demonstrating the importance of activating the cAMP/PKA pathway.

Due to PKA’s broad substrate specificity, it’s hard to say exactly which downstream targets are more significantly impacted by variants in the cAMP/PKA pathway. The neurodegenerative-linked L50R variant of *PRKAR1B* lies within the D/D domain which is responsible for PKA localization with AKAP’s in distinct subcellular compartments. Recent work from the Strack Lab indicate this variant impairs PKA C subunit compartmentalization (Glebov-McCloud et al., unpublished) and may prevent specific substrate phosphorylation through this intracellular targeting. However, there is a clear link between regulating transcription/translation of CRE promoted genes and neurodevelopmental disorders.

## Data Availability

Data sharing is not applicable to this article as no datasets were generated or analysed during the current study.

## Abbreviations

5-HT: 5-Hydroxytryptamine/serotonin
AC’s: Adenylyl cyclases
AD: Alzheimer’s disease
ADHD: Attention-deficit/hyperactivity disorder
AKAP’s: A-Kinase Associated Proteins
ASD: Autism spectrum disorder
ATP: Adenosine triphosphate
AVSD: Atrioventricular septal defect
BRET: Bioluminescence resonance energy transfer
C subunits: Catalytic subunits
CaMKII: Ca^2+^/Calmodulin-dependent protein kinase II
cAMP: Cyclic adenosine 3’,5’ monophosphate
CBP: CREB binding protein
CIT: Citron rho-interacting kinase
CNB domains: Cyclic nucleotide binding domains
CPA’s: Cortisol-producing adenomas
CRE: CAMP responsive element
CREB: CAMP response element-binding protein
D/D domain: Dimerization/Docking domain
EEG: Electroencephalography
EPSPs: Excitatory post-synaptic potentials
ERK: Extracellular signal-regulated kinase
FTD: Frontotemporal dementia
GDP: Guanosine diphosphate
GPCR’s: G protein-coupled receptors
GTP: Guanosine-5’-triphosphate
ID: Intellectual disability
KSR: Kinase suppressor of Ras
L-LTP: The late phase of long-term potentiation
LTD: Long-term depression
LTF: Long-term facilitation
LTM: Long-term memory
LTP: Long-term potentiation
MAPK: Mitogen-activated protein kinase
MASNS: Marbach-Schaaf Neurodevelopmental Syndrome
MRI: Magnetic resonance imaging
NDD: Neurodevelopmental disorder
NIFID: Neuronal intermediate filament inclusion disease
PDE: Phosphodiesterase
PKA: Protein Kinase A
R subunits: Regulatory subunits
R(AB): An inhibitory form of PKA RIα
SRE: Serum response element
SRF: Serum response factor
WT: Wild-type
